# Isolation and *in vitro* characterization of murine young-adult long bone skeletal progenitors

**DOI:** 10.3389/fendo.2022.930358

**Published:** 2022-08-01

**Authors:** Shauni Loopmans, Ingrid Stockmans, Geert Carmeliet, Steve Stegen

**Affiliations:** Laboratory of Clinical and Experimental Endocrinology, Department of Chronic Diseases and Metabolism, Katholieke Universiteit (KU) Leuven, Leuven, Belgium

**Keywords:** skeletal progenitor, hypoxia, osteoblast, adipocyte, chondrocyte, metaphysis, endosteum, cell isolation and expansion

## Abstract

Skeletal stem and progenitor cells (SSPCs) constitute a reservoir of bone-forming cells necessary for bone development, modeling and remodeling, as well as for fracture healing. Recent advances in tools to identify and isolate SSPCs have revealed that cells with multipotent properties are present not only in neonatal bone, but also in adult bone marrow and periosteum. The long bone metaphysis and endosteum have been proposed as an additional SSPC niche, although *in vitro* approaches to study their cellular and molecular characteristics are still limited. Here, we describe a comprehensive procedure to isolate and culture SSPCs derived from the metaphysis and endosteum of young-adult mice. Based on flow cytometry analysis of known SSPC markers, we found the presence of putative multipotent SSPCs, similar to neonatal bone tissue. *In vitro*, metaphyseal/endosteal SSPCs possess self-renewing capacity, and their multipotency is underscored by the ability to differentiate into the osteogenic and adipogenic lineage, while chondrogenic potential is limited. Expansion of metaphyseal/endosteal SSPCs under low oxygen conditions increases their proliferation capacity, while progenitor properties are maintained, likely reflecting their hypoxic niche *in vivo*. Collectively, we propose a validated isolation and culture protocol to study metaphyseal/endosteal SSPC biology *in vitro*.

## Introduction

In mammals, the skeleton functions as a supportive framework that also allows movement, contributes to hematopoiesis and calcium homeostasis, and participates in endocrine signaling (1). Bone homeostasis and repair are predominantly achieved by the concerted action of bone-resorbing osteoclasts and bone-forming osteoblasts. Similar to osteoclasts that descend from hematopoietic stem cells, the mesenchymal lineage consists of a complex hierarchy of self-renewing, multipotent stem cells and more lineage-committed progenitors (1). Skeletal stem and progenitor cells (SSPCs) are critical for bone health, because they are a continuous source of mesenchymal cell types, including osteoblasts, chondrocytes and other stromal cells. Accordingly, perturbations in SSPC number, function or fate are associated with skeletal pathologies such as osteoporosis, osteoarthritis and fracture non-unions that manifestly affect patient quality of life and impose a significant burden on the health care system.

SSPCs were classically defined by *in vitro* characteristics, including plastic adherence, the potential to generate fibroblast-like colonies and the ability to differentiate into osteoblasts, chondrocytes and adipocytes (2). Recent methodological advances in cell lineage tracing, flow cytometry and single-cell transcriptomics have improved our understanding of SSPC location, fate and function *in vivo* (3–6). These studies clearly show that the skeleton comprises several anatomically distinct SSPC niches, including the bone marrow, the periosteum and the articular and epiphyseal cartilage (5). SSPCs from these different skeletal regions share overlapping properties, including self-renewing potential and multipotency, although lineage specification may differ as well as surface marker expression (3). In addition, the metaphysis and endosteum have been proposed as putative SSPC niches, based on the presence of cells that express SSPC markers, which include, but are not limited to, GLI family zinc finger 1 (Gli1), Gremlin 1 (Grem1), Leptin receptor (LepR), Nestin-GFP, Platelet-derived growth factor receptor α (PDGFRα) and PDGFRβ (7–13). Still, these metaphyseal and endosteal SSPC populations remain poorly characterized.

A generally accepted approach to study SSPC biology is the use of *in vitro* cultures, as it allows to investigate SSPC properties, including cell growth, self-renewing potential and multipotency. Given that SSPCs at different skeletal locations may differ in specific properties, it is critical that the used protocol specifically isolates the SSPCs of the region of interest. Protocols to isolate and culture SSPCs from specific skeletal locations, including the bone marrow and periosteum, have previously been established (14–18), but a validated protocol to isolate SSPCs specifically from the long bone metaphysis and endosteum with a minimal number of contaminating cells is lacking.

We here describe a detailed procedure to isolate SSPCs from the long bone trabecular and endosteal regions of young-adult mice. Based on previously described SSPC markers (19), we show that metaphyseal and endosteal bone contains a population of skeletal progenitors with *in vitro* self-renewing capacity and the ability to differentiate into the osteogenic and adipogenic lineage, whereas their chondrogenic potential is limited. In addition, we show that *in vitro* expansion of metaphyseal/endosteal SSPCs under low oxygen conditions favors cell growth without loss of SSPC properties. In conclusion, we established a highly feasible and consistent protocol to isolate and culture long bone metaphyseal/endosteal SSPCs, which could serve as a useful model to study their cellular and molecular properties *in vitro.*

## Materials and methods

### Animals

All experiments were performed using male and female C57BL/6J mice (100% C57BL/6 background). Mice were housed and bred in conventional conditions (Proefdierencentrum Leuven, Belgium).

### Cell isolation and culture

#### Metaphyseal/endosteal long bone skeletal stem and progenitor cells (SSPCs) from young-adult mice

**Figure 1:**
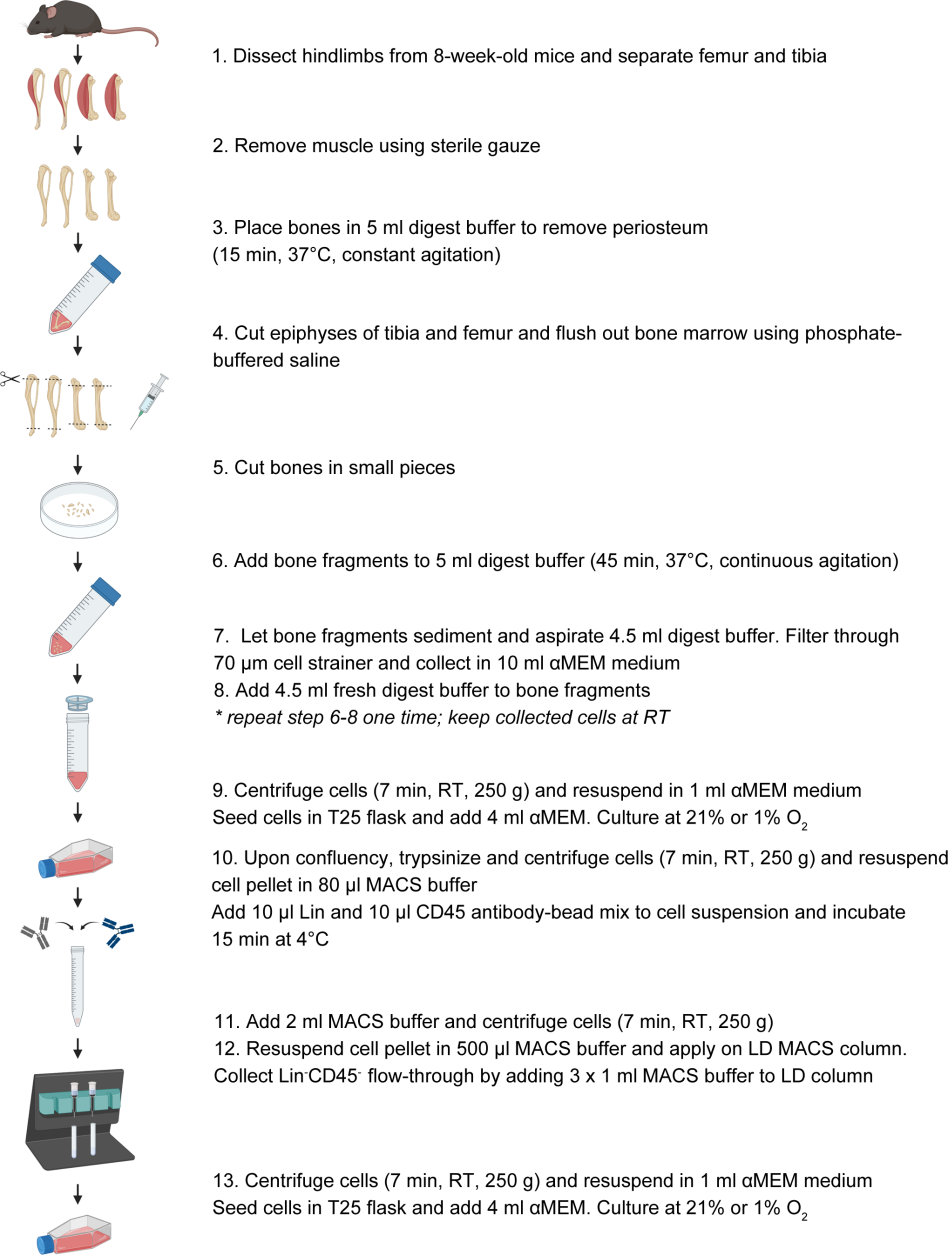
Schematic overview of the experimental setup to isolate long bone metaphyseal/endosteal SSPCs from young-adult mice.

To isolate metaphyseal and endosteal long bone SSPCs, tibias and femurs were dissected from 6-to-8-week old mice (**Figure 1**), and muscle and connective tissue were removed under sterile conditions. Bones were incubated in 2 ml digest buffer (3 mg/ml collagenase type II, 4 mg/ml dispase in α minimal essential medium (αMEM) GlutaMAX™ containing 100 units/ml penicillin, 50 µg/ml streptomycin (all from Gibco; ThermoFisher Scientific, Belgium) for 15 min at 37°C with constant agitation to remove the periosteum. The periosteal cell digest was discarded, bones were washed with phosphate-buffered saline (PBS; Gibco), epiphyses were removed and bones were flushed using PBS. Subsequently, bone shafts were cut into smaller pieces and incubated in 2 ml digest buffer for two consecutive digests of 45 min at 37°C under constant agitation. The cell suspension, obtained after each digest, was passed through a 70 µm cell strainer, washed with culture medium (αMEM GlutaMAX™ containing 100 units/ml penicillin, 50 µg/ml streptomycin and 10% fetal bovine serum (FBS; Gibco)) and kept at RT. The two cell digests were combined, centrifuged (250 g for 7 min at RT) and cells were used either for flow cytometry analysis (as detailed in the ‘Flow Cytometry’ methods section), or for *in vitro* expansion.

For *in vitro* expansion, isolated metaphyseal and endosteal SSPCs were pooled per two mice and seeded in T25 culture flasks. Cells were expanded in either normoxia (21% O_2_) or hypoxia (1% O_2_) at 37°C and 5% CO_2_. Upon 80% confluency (i.e. after approximately one week of culture), cells were trypsinized (Trypsin-EDTA 0.05%; Gibco) and centrifuged at 250 g for 7 min at RT. The cell pellet was resuspended in 100 µl magnetic-activated cell sorting (MACS) buffer (PBS containing 2% FBS) to which Direct Lineage (Lin) and CD45 antibody-microbead solutions were added (both 1:10 dilution; both from Miltenyi biotec, Germany) and incubated for 15 min at 4°C. After antibody incubation, cells were washed with 2 ml MACS buffer, centrifuged (250 g for 7 min at 4°C) and resuspended in 500 µl MACS buffer before loading onto a LD MACS column (Miltenyi biotec, Germany). The flow-through Lin^-^CD45^-^ fraction was collected (3 times wash with 1 ml MACS buffer), centrifuged (250 g for 7 min at 4°C), and resuspended in culture medium prior to seeding in a T25 flask. Cells were expanded in either normoxia (21% O_2_) or hypoxia (1% O_2_) at 37°C and 5% CO_2_. Upon 80% confluency (i.e. after approximately one week of culture), cells were counted using the LUNA™ Automated Cell Counter (Logos Biosystems; Westburg, Belgium) and seeded for experiments.

#### SSPCs from neonatal long bones

**Figure 2:**
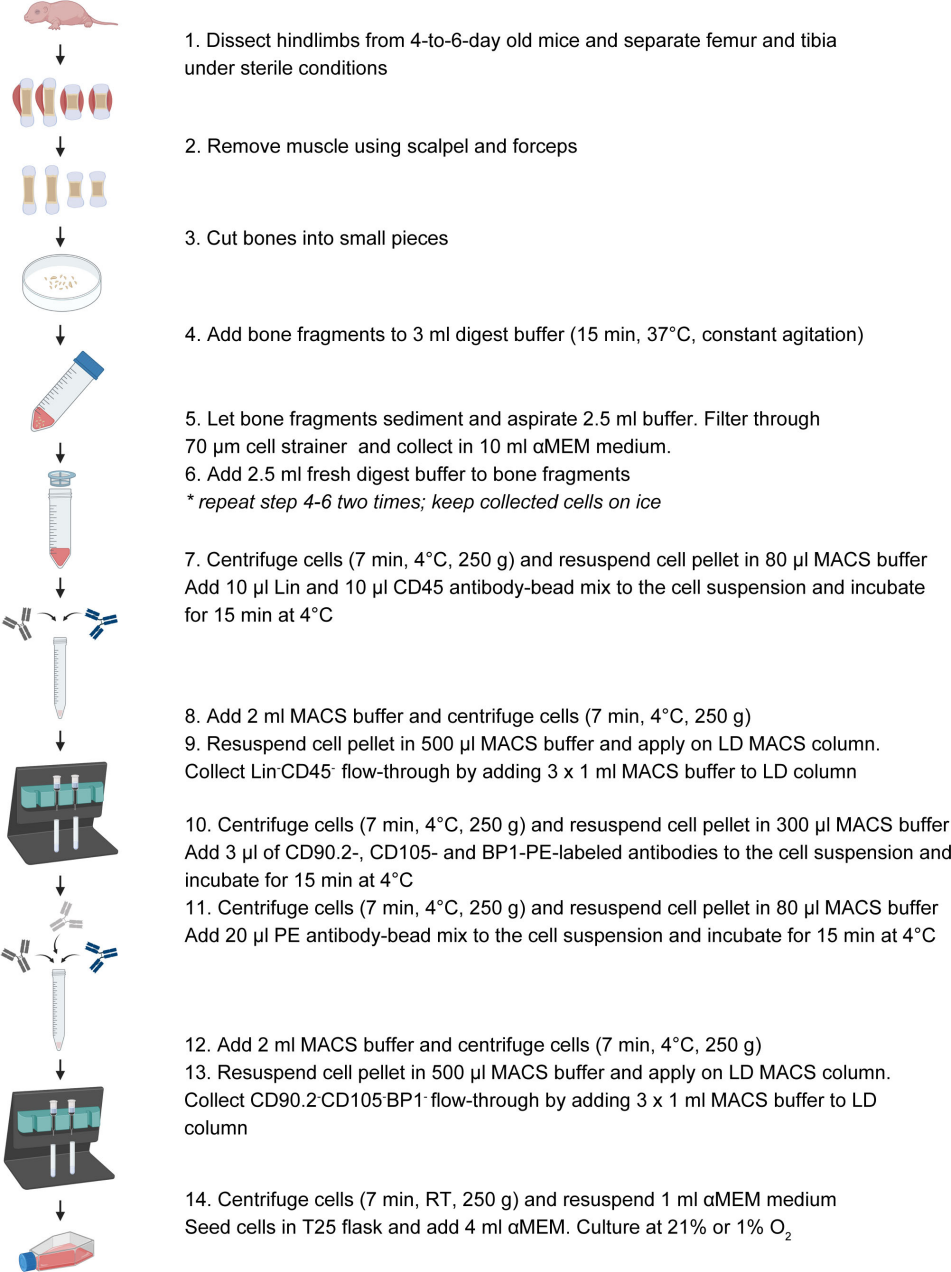
Schematic overview of the experimental setup to isolate SSPCs from neonatal long bones.

Neonatal long bone SSPCs were isolated from tibias and femurs of 4-to-6-day-old male and female mice (**Figure 2**). First, muscle and connective tissue were removed under sterile conditions and bones were cut into small pieces. Next, bone fragments were incubated in 3 ml digest buffer (3 mg/ml collagenase type II, 4 mg/ml dispase and 100 units/ml DNAse 1 (Roche; Merck, Belgium) in αMEM GlutaMAX™ containing 100 units/ml penicillin, 50 µg/ml streptomycin) for three consecutive digests of 15 min at 37°C with constant agitation. After each digest, 2.5 ml cell suspension was passed through a 70-µm cell strainer, collected in culture medium (αMEM GlutaMAX™ containing 100 units/ml penicillin, 50 µg/ml streptomycin and 10% FBS) and kept on ice. Cells combined of the three digests were centrifuged (250 g for 7 min at 4°C) and prepared either for flow cytometry analysis (as detailed in the ‘Flow Cytometry’ methods section), or for *in vitro* expansion.

For *in vitro* expansion, the cell pellet was resuspended in 100 µl MACS buffer containing Direct Lin and CD45 antibody-microbead solutions (both 1:10 dilution) and incubated for 15 min at 4°C. After antibody incubation, cells were washed with 2 ml of MACS buffer, centrifuged (250 g for 7 min at 4°C) and resuspended in 500 µl MACS buffer before loading onto a LD MACS column. The Lin^-^CD45^-^ flow-through was collected (3 times wash with 1 ml MACS buffer), centrifuged (250 g for 7 min at 4°C), and resuspended in 300 µl MACS buffer containing CD90.2-PE (eBioscience™; ThermoFisher Scientific, Belgium), CD105-PE (eBioscience™) and BP1-PE-conjugated (eBioscience™, Belgium) antibodies (all 1:100 dilution). After 15 min incubation at 4°C, cells were washed with 2 ml MACS buffer and centrifuged (250 g for 7 min at 4°C). The cell pellet was resuspended in 100 µl MACS buffer containing PE antibody-microbead solution (1:5 dilution; Miltenyi biotec, Germany) for 15 min at 4°C. Next, cells were washed with 2 ml of MACS buffer, centrifuged (250 g for 7 min at 4°C), resuspended in 500 µl MACS buffer and loaded onto an LD MACS column. The PE negative (CD90.2^-^CD105^-^BP1^-^) flow-through was collected (3 times wash with 1 ml MACS buffer), centrifuged (250 g for 7 min at RT), resuspended in culture medium and seeded in T25 flasks. Cells isolated from two mice were pooled per T25. Neonatal SSPCs were expanded in either normoxia (21% O_2_) or hypoxia (1% O_2_) at 37°C and 5% CO_2_. Upon 80% confluency (i.e. after approximately one week of culture), cells were counted using the LUNA™ Automated Cell Counter and seeded for experiments.

### Flow cytometry

#### Freshly isolated cells from metaphyseal/endosteal and neonatal bone

Bone cells were isolated according to the protocol as described above, without MACS-mediated cell enrichment. Cells were resuspended in Hank’s Buffered Salt Solution (HBSS; Gibco) containing 2% FBS and incubated with antibodies against BP1, CD31, CD45, CD51, CD90.2, CD105, CD200 and Ter119 for 10 min at 4°C. Streptavidin-labeled antibodies were used as secondary antibody when appropriate (**Table 1**). Unlabeled and single-fluorophore samples were used as gating controls.

**Table 1:** List of antibodies and microbeads used for flow cytometry analysis or MACS depletion.

	Antigen	Conjugate	Supplier	Catalog number	Dilution	Clone ID
Primary antibodies	BP1	PE	eBioscience	12-5891-81	1:100	6C3
	CD31	APC	eBioscience	17-0311-80	1:500	390
	CD45	APC	eBioscience	17-0451-83	1:500	30-F11
	CD51	Biotin	eBioscience	13-0512-82	1:100	RMV-7
	CD90.2	PE	eBioscience	12-0903-81	1:100	30-H12
	CD105	PE	eBioscience	12-1051-81	1:100	MJ7/18
	CD200	FITC	ThermoFisher	MA5-17980	1:100	OX-90
	Ter119	APC	eBioscience	17-5921-83	1:200	TER-119
Secondary antibodies	Streptavidin	Pacific Blue	ThermoFisher	S11222	1:100	-
Microbeads	CD45	-	Miltenyi Biotech	130-052-301	1:10	-
	Direct Lineage	-	Miltenyi Biotech	130-110-470	1:10	-
	PE	-	Miltenyi Biotech	130-105-639	1:5	-

#### Cultured SSPCs from metaphyseal/endosteal and neonatal bone

Metaphyseal/endosteal and neonatal SSPCs (MACS depleted and expanded as described above) were trypsinized (Trypsin-EDTA 0.05%) and stained with antibodies against CD31, CD45, CD51 and Ter119 in HBSS with 2% FBS for 10 min at 4°C. Streptavidin-labeled antibodies were used as secondary antibody when appropriate (**Table 1**). Unlabeled and single-fluorophore samples were used as gating controls.

Flow cytometry was performed on the BD FACSCanto II (BD Biosciences, Belgium) and data was analyzed using the Kaluza software (Beckman Coulter, CA, USA).

### Cellular properties

#### Fibroblastic colony-forming unit (CFU-F) assay

The CFU-F assay was performed in normoxic conditions as described before (20). Briefly, metaphyseal/endosteal and neonatal SSPCs were seeded at a density of 10 cells/cm^2^ in Dulbecco’s Modified Eagle Medium (DMEM; Gibco) containing 25 mM glucose, 2 mM glutamine and 15% FBS. Medium was changed twice per week and at day 10, colonies were either stained using 1% methylene blue (Sigma-Aldrich; Merck, Belgium) in 10 mM borate buffer, or trypsinized (Trypsin-EDTA 0.05%) and reseeded at CFU density for secondary CFU formation.

Colonies containing at least 20 cells were counted, and primary and secondary CFU efficiency was calculated as the percentage of colonies per seeded cell number.

#### Osteogenic differentiation

Osteogenic differentiation was performed in normoxic conditions as described by Stegen et al. (21). Cells were seeded in culture medium at a density of 25,000 cells/cm^2^. Upon confluency, culture medium was switched to osteogenic differentiation medium (culture medium containing 50 µg/ml L-ascorbic acid and 10 mM β-glycerophosphate; both from Sigma-Aldrich; Merck, Belgium). Osteogenic differentiation medium was refreshed twice per week and mineralization was visualized after staining with 0.1% Alizarin Red (Sigma-Aldrich) pH 6.2 at day 21 of differentiation. To quantify staining intensity, Alizarin Red was extracted using 10% acetic acid. Samples were heated to 85°C for 10 minutes, followed by 5 minutes incubation on ice. After centrifugation (20,000 g for 15 min at RT), supernatant was collected and 10% NH_4_OH was added prior to measuring absorbance at 405 nm (Synergy H1 spectrophotometer; BioTek, VT, USA).

#### Adipogenic differentiation

Adipogenic differentiation was performed in normoxic conditions as described previously (14). Cells were seeded in culture medium at a density of 25,000 cells/cm^2^. The next day, medium was switched to adipogenic differentiation medium (culture medium containing 50 µM indomethacin, 500 nM 3-isobutyl-1-methylxanthine, 10 nM dexamethasone and 10 µg/ml insulin from bovine pancreas all from Sigma-Aldrich). Adipogenic differentiation medium was refreshed twice per week and lipid droplets were stained at day 14 using 0.2% Oil Red O (Sigma-Aldrich) in 60% isopropanol. Staining intensity was quantified after Oil Red O extraction using 100% isopropanol and measurement at 515 nm (Synergy H1 spectrophotometer).

#### Chondrogenic differentiation

Chondrogenic differentiation was performed in normoxic conditions as described before (22). Briefly, 125,000 cells were drop-seeded in 10 µl culture medium. After cell attachment (1 hour at 37°C), chondrogenic differentiation medium (αMEM GlutaMAX™ containing 100 units/ml penicillin, 50 µg/ml streptomycin, 1% FBS, 50 µg/ml L-ascorbic acid, 10 ng/ml recombinant human transforming growth factor-β1 (rhTGF-β1; PeproTech, UK) and 20 µM Y-27632 (Axon Medchem, Netherlands)) was added to the wells. Chondrogenic differentiation medium was refreshed at day 3 and day 6, and glycosaminoglycans were stained at day 9 using 1% Alcian Blue 8GX (Sigma-Aldrich) in 0.1N HCl. Staining intensity was quantified after Alcian Blue extraction using 6M guanidine hydrochloride and measurement at 620 nm (Synergy H1 spectrophotometer).

### Statistical analysis

Data are shown as mean ± SD and analyzed using Student’s *t*-test or two-way ANOVA with Tukey *post hoc* test (GraphPad Prism software). All experiments were independently repeated at least twice, with *n* values representing the total number of biological replicates. Cells isolated from two mice were pooled per biological sample. Differences were considered statistically significant at p<0.05.

### Figure artwork

Schematics included in the figures were created using BioRender.

## Results

### Metaphyseal/endosteal bone contains cells that express skeletal stem and progenitor cell surface markers

**Figure 3:**
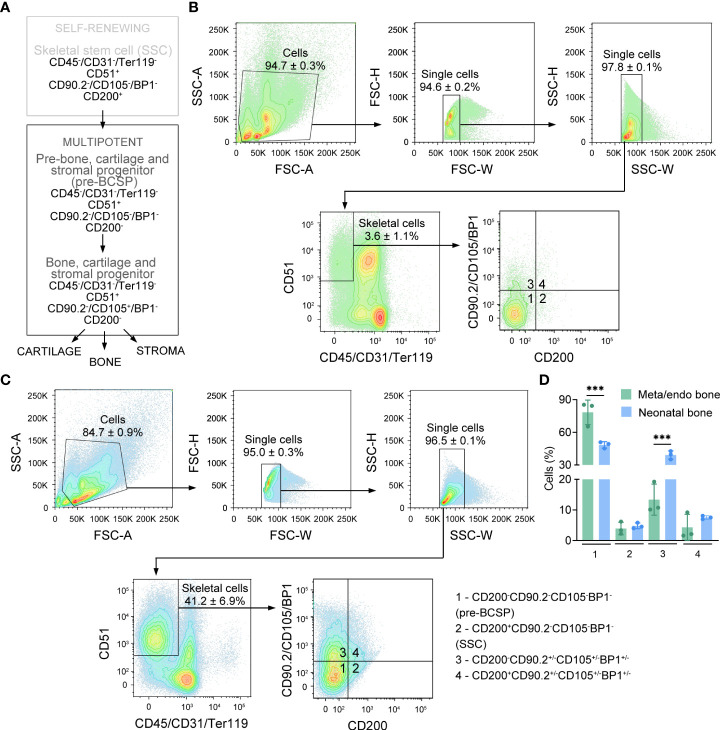
Freshly isolated metaphyseal/endosteal bone cells express surface markers of putative stem and progenitor cells. **(A)** Schematic representation of the mouse skeletal stem cell tree (adapted from (19)). **(B)** Contour plots showing the gating strategy used for flow cytometry-based quantification of different skeletal cell subpopulations in freshly isolated metaphyseal/endosteal cells (n=3). **(C)** Contour plots showing the gating strategy used for flow cytometry-based quantification of different skeletal cell subpopulations in freshly isolated neonatal cells (n=3). **(D)** Quantification of different cell subpopulations in metaphyseal/endosteal and neonatal SSPCs based on CD200/CD90.2/CD105/BP1 expression (n=3). SSC is skeletal stem cell, pre-BCSP is pre-bone, cartilage and stromal progenitor. Data are shown as mean ± SD. ***p<0.001 (Two-way ANOVA with Tukey’s multiple comparison test).

Bone and bone marrow stromal cells represent a heterogenous mixture of different cell populations with distinct molecular and functional properties (13, 23–25). To characterize the cellular subpopulations that comprise the bone metaphysis/endosteum in young-adult mice, we performed flow cytometry analysis on freshly isolated cells using validated stem and progenitor surface markers expressed by SSPCs from neonatal long bones [**Figure 3A**; (19)]. To isolate metaphyseal/endosteal bone cells, we removed epiphyses from long bones, flushed out the bone marrow and chopped bone shafts into smaller pieces before enzymatic digest. For neonatal bone cells, long bones were cleaned of muscle and connective tissue, and the entire bones (containing epiphyses and bone marrow) were cut into smaller pieces, followed by enzymatic digest. To discriminate between skeletal versus non-skeletal cells, we first assessed differential expression of CD45, CD31, Ter119 and CD51, which are surface markers expressed by hematopoietic (CD45, Ter119), vascular (CD31) and skeletal cells (CD51). We noticed that the frequency of CD51^+^CD45^-^CD31^-^Ter119^-^ cells (hereafter referred to as skeletal cells) was lower in metaphyseal/endosteal bone compared to neonatal bone (**Figures 3B**–**D**; 3.6% *vs*. 41.2% gated on single cell population), and this observation is likely explained by the relatively high number of hematopoietic cells in adult bone marrow, even after flushing.

The CD51^+^CD45^-^CD31^-^Ter119^-^ skeletal cell population consists of distinct stem and progenitor cell pools, that differ in their differentiation stage and lineage commitment. To compare the obtained metaphyseal/endosteal population to a validated source of SSPCs, we used SSPCs isolated from neonatal mice, based on the cell surface markers described by Chan et al. (19). Self-renewing, multipotent skeletal stem cells (SSCs) express CD200, but are negative for CD90.2, CD105 and BP1 (**Figure 3A**). These SSCs differentiate into more lineage-restricted progenitor cells called pre-bone, cartilage and stromal progenitors (pre-BCSPs; CD200^-^CD90.2^-^CD105^-^BP1^-^), and bone, cartilage and stromal progenitors (BCSPs; CD200^-^CD90.2^-^CD105^+^BP1^-^). We found that the majority of the skeletal cell pool in metaphyseal/endosteal bone were pre-BCSPs, a population that was significantly larger compared to neonatal bone (**Figures 3B**–**D**; 78.4% *vs*. 48.5% CD51^+^CD45^-^CD31^-^Ter119^-^CD200^-^CD90.2^-^CD105^-^BP1^-^ cells respectively). This difference might be explained by the fact that for the isolation of neonatal SSPCs, we used total bone including growth plate tissue, which contains CD105-expressing chondrocytes (19, 26). On the other hand, around 5% of the skeletal cell pool in both metaphyseal/endosteal and neonatal bone expressed SSC-related surface markers (**Figures 3B**–**D**; 3.9% CD51^+^CD45^-^CD31^-^Ter119^-^CD200^+^CD90.2^-^CD105^-^BP1^-^ cells in metaphyseal/endosteal bone *vs*. 4.8% in neonatal bone). Notably, because we used CD90.2, CD105 and BP1 antibodies that were labeled with the same fluorophore, it was not possible to further characterize the different progenitor subpopulations. Finally, because our isolation protocol was not able to distinguish between SSPCs isolated from the metaphysis and the endosteum, this progenitor cell population will hereafter be referred to as ‘metaphyseal/endosteal SSPCs’.

Taken together, using flow cytometry-based analysis, we could show that the skeletal fraction of young-adult metaphyseal/endosteal bone consists of a heterogenous cell population comprising putative self-renewing, multipotent SSCs and pre-BCSPs.

### Culturing skeletal lineage-enriched metaphyseal/endosteal SSPCs in hypoxic conditions increases cell number

We next aimed to set up an *in vitro* cell culture model to analyze the properties of metaphyseal/endosteal SSPCs, focusing on (i) using a purified SSPC population and (ii) mimicking the low oxygen environment of the *in vivo* SSPC niche. Indeed, our flow cytometry analysis indicated that only a minor fraction of the freshly isolated cells from the bone metaphysis and endosteum consists of skeletal cells expressing stem and progenitor cell-related surface markers, whereas more than 90% are hematopoietic and/or vascular cells (**Figure 3B**) that could potentially hinder functional analyses (27). Therefore, we depleted Lineage (Lin) and CD45-expressing cells by MACS and this Lin^-^CD45^-^ enriched cell population is hereafter referred to as metaphyseal/endosteal SSPCs. We opted to deplete Lin^+^CD45^+^ cells after *in vitro* expansion for one passage, because of the very low number of skeletal cells in the freshly isolated metaphyseal/endosteal cell pool (**Figure 3B**) and the fact that MACS depletion immediately after isolation impaired cell growth (data not shown). For means of comparison, we used Lin/CD45-depleted neonatal long bone cells, which were further enriched for cells that were negative for CD90.2, CD105 and BP1 (hereafter referred to as neonatal SSPCs). The rationale for this approach was based on the observation that only ~50% of skeletal cells in neonatal long bones were pre-BCSPs, whereas this fraction was significantly higher in the skeletal cell fraction in metaphyseal/endosteal bone (~80%).

**Figure 4:**
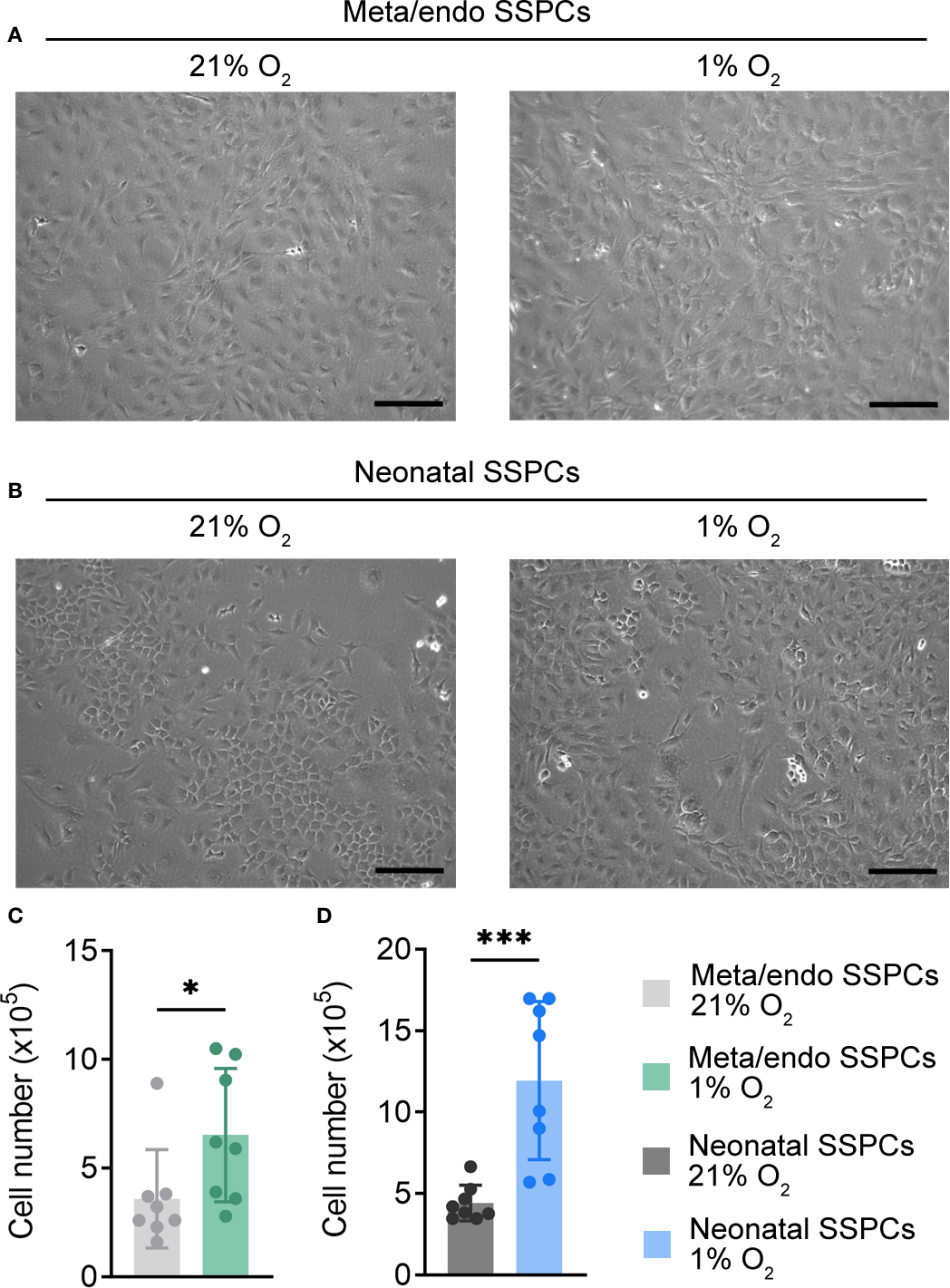
Expansion of neonatal and metaphyseal/endosteal SSPCs in hypoxia increases cell number. **(A)** Microscopic image of metaphyseal/endosteal SSPCs expanded in 21% O_2_ and 1% O_2_. Scale bar is 100 µm. Images were taken after one week of culture. **(B)** Microscopic image of neonatal SSPCs expanded in 21% O_2_ and 1% O_2_. Scale bar is 100 µm. Images were taken after one week of culture. **(C, D)** Absolute cell number of metaphyseal/endosteal **(C)** and neonatal SSPCs **(D)** after expansion in 21% or 1% O_2_ (n=8). Data are shown as mean ± SD. *p<0.05, ***p<0.001 (Student’s *t*-test).

Several studies have shown that *in vitro* expansion of skeletal progenitors in low oxygen conditions is beneficial for cell growth and for maintaining their typical progenitor properties, as it likely mimics the hypoxic bone microenvironment (28–30). We therefore cultured metaphyseal/endosteal SSPCs in hypoxia (1% O_2_) and investigated its effect on cell morphology and number, using neonatal SSPCs as a reference cell type (**Figures 4A–D**). Similar to neonatal SSPCs, hypoxia-expanded metaphyseal/endosteal SSPCs displayed a typical spindle-shaped morphology and showed a two-fold increase in cell growth when compared to normoxic conditions (21% O_2_).

**Figure 5:**
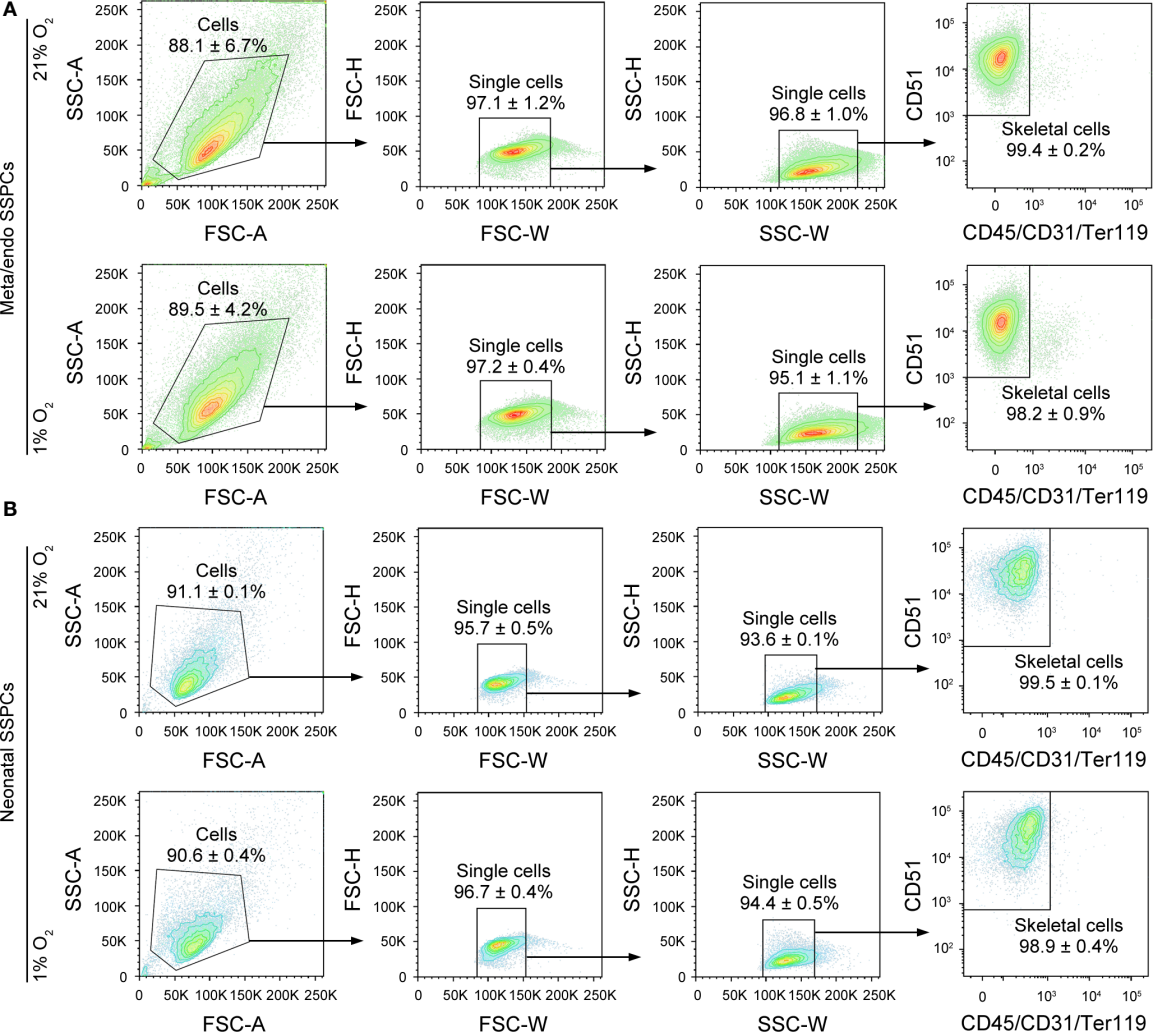
Flow cytometry-based validation of depleting the contaminating CD45^+^/CD31^+^/Ter119^+^ cell population in cultured metaphyseal/endosteal and neonatal cells. **(A)** Contour plots showing the gating strategy used for flow cytometry-based quantification of the percentage of skeletal cells (CD51^+^CD45^-^CD31^-^Ter119^-^) after depletion of CD45^-^CD31^-^Ter119^-^ and expansion of purified metaphyseal/endosteal SSPCs in 21% O_2_ (upper panels) and in 1% O_2_ (lower panels) (n=3). **(B)** Contour plots showing the gating strategy used for flow cytometry-based quantification of the percentage of skeletal cells (CD51^+^CD45^-^CD31^-^Ter119^-^) after depletion of CD45^-^CD31^-^Ter119^-^ and expansion of neonatal SSPCs in 21% O_2_ (upper panels) and in 1% O_2_ (lower panels) (n=3). Data are shown as mean ± SD.

Finally, we determined by flow cytometry whether metaphyseal/endosteal SSPCs maintain their typical skeletal lineage marker expression after *in vitro* expansion. Flow cytometry analysis of cultured cells prior to MACS depletion showed approximately 45% of metaphyseal/endosteal and 5% of neonatal cells were positive for CD45, CD31 and/or Ter119 (**Supplementary Figure 1A–D**). After MACS-mediated depletion, we found that almost all cultured metaphyseal/endosteal SSPCs were CD51^+^CD45^-^CD31^-^Ter119^-^ (99.4%), and that there was no differential effect of expanding these cells in hypoxic or normoxic conditions (**Figure 5A**). Similar data were obtained when using neonatal SSPCs (**Figure 5B**). Finally, although removing CD45, CD31 and/or Ter119 had no effect on CD105 and CD200 marker expression in metaphyseal/endosteal SSPCs (**Supplementary Figure 2A**), we noticed that this strategy was necessary for proper osteogenic differentiation (**Supplementary Figure 2B**).

Together, these data indicate that (time-dependent) plastic adherence is not sufficient to remove hematopoietic and vascular cells from the cell population obtained from the metaphyseal/endosteal bone region. An extra step using cell surface markers to remove these contaminating cells, significantly enriches for skeletal cells and improves their osteogenic differentiation capacity. Moreover, expanding metaphyseal/endosteal SSPCs in low oxygen conditions is beneficial for cell growth, resulting in a sufficiently high cell number that can readily be used for subsequent *in vitro* studies.

### Metaphyseal/endosteal SSPCs display *in vitro* self-renewing capacity and multipotency

**Figure 6:**
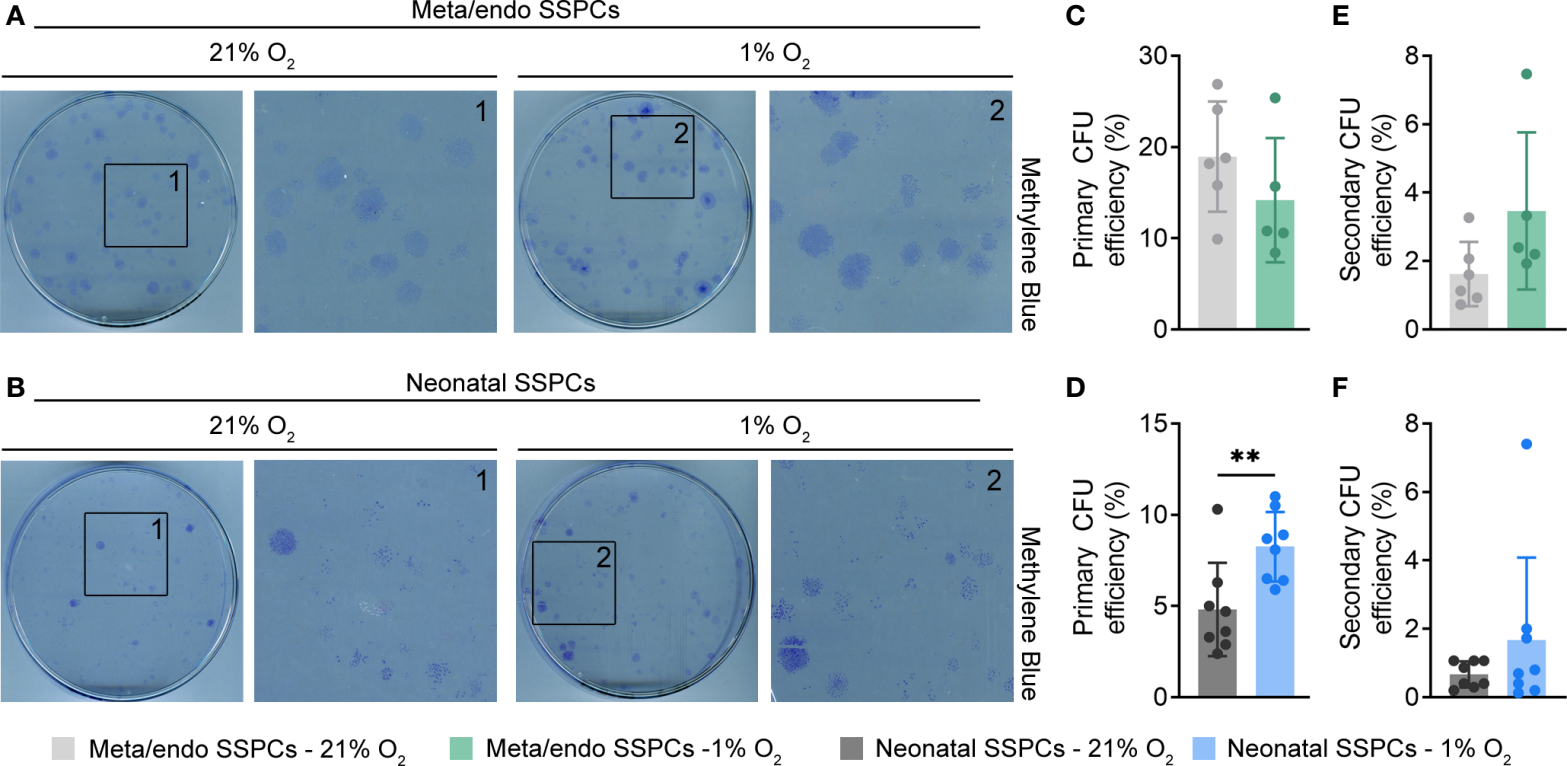
Metaphyseal/endosteal SSPCs display higher *in vitro* self-renewing capacity compared to neonatal SSPCs. **(A)** Methylene Blue staining of primary colonies formed by metaphyseal/endosteal SSPCs expanded in 21% or 1% O_2_ (n=5-6). Boxed areas are magnified. **(B)** Methylene Blue staining of primary colonies formed by neonatal SSPCs expanded in 21% or 1% O_2_ (n=8). Boxed areas are magnified. **(C)** Primary colony-forming unit (CFU) efficiency of metaphyseal/endosteal SSPCs expanded in 21% or 1% O_2_ (n=5-6). **(D)** Primary CFU efficiency of neonatal SSPCs expanded in 21% or 1% O_2_ (n=8). **(E)** Secondary CFU efficiency of metaphyseal/endosteal SSPCs expanded in 21% or 1% O_2_ (n=5-6). **(F)** Secondary CFU efficiency of neonatal SSPCs expanded in 21% or 1% O_2_ (n=8). Data are shown as mean ± SD. **p<0.01 (Student’s *t*-test).

Hypoxic expansion is thus beneficial for metaphyseal/endosteal SSPC growth. We next determined whether cultured metaphyseal/endosteal SSPCs also maintain their putative stem and progenitor properties *in vitro*, by investigating their colony-forming potential, their self-renewal and multipotency. Using the CFU-F assay, we noticed that metaphyseal/endosteal SSPCs gave rise to a higher frequency of colony formation compared to neonatal SSPCs, and that hypoxic expansion did improve the colony-forming potential of neonatal SSPCs, but not of metaphyseal/endosteal SSPCs (**Figures 6A–D**).

We next questioned whether metaphyseal/endosteal SSPCs were capable of *in vitro* self-renewal by testing their ability to serially generate CFUs. To this end, primary colonies were harvested, reseeded at colony-forming density and the CFU-F assay was repeated. Passaging primary metaphyseal/endosteal SSPC colonies resulted in the formation of secondary colonies with similar morphology to those of primary colonies (data not shown) and, similar as observed with primary CFU formation, at a higher efficiency compared to neonatal SSPCs (**Figures 6E**, **F**). Hypoxic expansion did not manifestly affect secondary colony formation by either metaphyseal/endosteal or neonatal SSPCs. Thus, the metaphyseal/endosteal SSPC pool comprises cells with *in vitro* self-renewal features.

**Figure 7:**
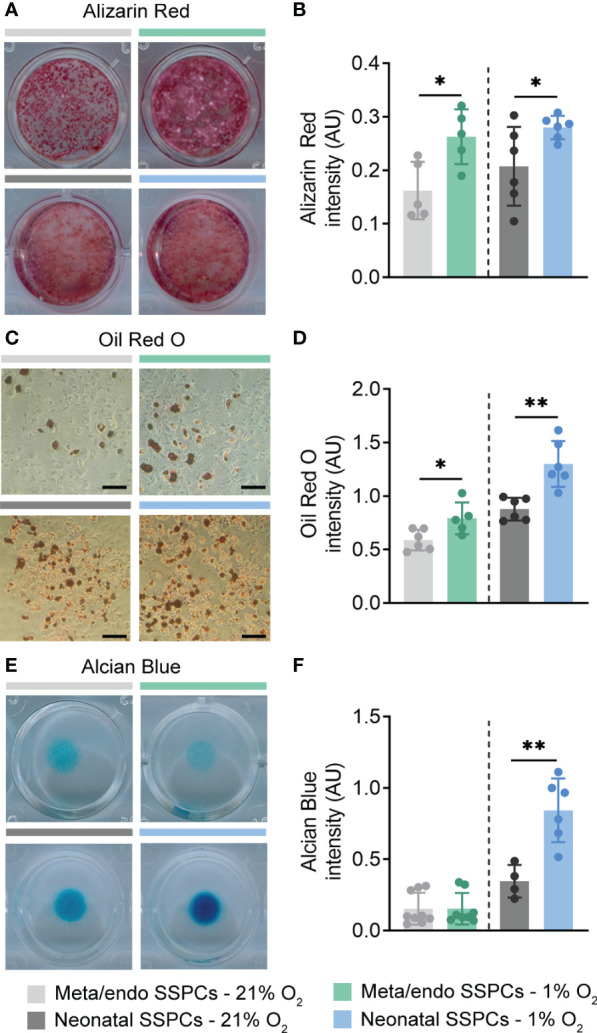
Metaphyseal/endosteal SSPCs differentiate into osteoblasts and adipocytes, but exhibit limited chondrogenic potential. **(A)** Alizarin Red staining at day 21 of osteogenic differentiation of metaphyseal/endosteal (upper panels) and neonatal SSPCs (lower panels), expanded in 21% or 1% O_2_ (n=5-6). **(B)** Quantification of Alizarin Red staining intensity at day 21 of osteogenic differentiation (n=5-6). **(C)** Oil Red O staining at day 14 of adipogenic differentiation of metaphyseal/endosteal (upper panels) and neonatal SSPCs (lower panels), expanded in 21% or 1% O_2_ (n=5-6). Scale bar is 100 µm. **(D)** Quantification of Oil Red O staining intensity at day 14 of adipogenic differentiation (n=5-6). **(E)** Alcian Blue staining of micromass cultures at day 9 of chondrogenic differentiation of metaphyseal/endosteal (upper panels) and neonatal SSPCs (lower panels), expanded in 21% or 1% O_2_ (n=4-9). **(F)** Quantification of Alcian Blue staining intensity at day 9 of chondrogenic differentiation (n=4-9). Data are shown as mean ± SD. *p<0.05, **p<0.01 (Student’s *t*-test).

Another known feature of SSPCs is the potential to differentiate into the osteogenic, adipogenic and chondrogenic lineage (3). To test their multipotent potential, we first cultured metaphyseal/endosteal SSPCs in osteogenic differentiation medium containing β-glycerophosphate and L-ascorbic acid in normoxic conditions. At day 21 of osteogenic induction, we observed clear formation of mineralized nodules, stained by Alizarin Red, and differentiation was comparable between the two sources of SSPCs (**Figures 7A**, **B**). Expansion of metaphyseal/endosteal and neonatal SSPCs in 1% O_2_ improved osteogenic differentiation capacity in both cell types (**Figures 7A**, **B**).

Next, we examined whether we could induce adipogenesis by culturing SSPCs in 21% O_2_ in the presence of indomethacin, 3-isobutyl-1-methylxanthine, dexamethasone and insulin. At day 14 of adipogenic differentiation, we observed the formation of lipid droplets in both metaphyseal/endosteal and neonatal SSPC cultures, as evidenced by Oil Red O staining (**Figures 7C**, **D**). Again, initial expansion under low oxygen conditions increased lipid droplet formation in both SSPC populations (**Figures 7C**, **D**).

Finally, we investigated the chondrogenic potential of metaphyseal/endosteal SSPCs in normoxic conditions. To this end, cells were drop-seeded and cultured as micromasses in the presence of L-ascorbic acid, TGF-β and ROCK inhibitor. Neonatal SSPCs displayed strong chondrogenic potential, which was even further increased when these cells were expanded in 1% O_2_, as evidenced by Alcian Blue staining at differentiation day 9 (**Figures 7E**, **F**). In contrast, chondrogenic capacity of metaphyseal/endosteal SSPCs was very limited and was not stimulated by hypoxic expansion (**Figures 7E**, **F**).

Collectively, our data show that metaphyseal/endosteal SSPCs possess the ability to self-renew and differentiate into the osteogenic and adipogenic lineage *in vitro*, and that this multipotent potential was improved when cells were expanded in 1% O_2_.

## Discussion

SSPCs represent a (self-renewing) pool of progenitor cells that are essential for skeletal development, homeostasis and repair. Recent studies have identified several SSPC niches localized at different skeletal sites, including the long bone metaphysis and endosteum, although insight in the molecular properties of metaphyseal/endosteal SSPCs is still limited. In this study, we optimized an isolation procedure and culture conditions to study murine metaphyseal/endosteal SSPCs *in vitro.*

Different phenotypic markers, expressed either at the cell surface or intracellularly, have been used to identify SSPCs in the murine skeleton. In our study, we have opted to characterize the cellular heterogeneity of the metaphyseal/endosteal skeletal cell pool using validated surface markers described by Chan et al. (19). The rationale for this approach was that (i) surface markers allow the identification and isolation of putative metaphyseal/endosteal SSPCs by flow cytometry without the use of lineage reporter mice and (ii) we could compare them to the well-characterized SSPC population derived from neonatal mice using the same markers. Flow cytometry analysis revealed the presence of putative skeletal stem cells (CD51^+^CD45^-^CD31^-^Ter119^-^CD200^+^CD90.2^-^CD105^-^BP1^-^) and multipotent pre-progenitors (CD51^+^CD45^-^CD31^-^Ter119^-^CD200^-^CD90.2^-^CD105^-^BP1^-^) in the freshly isolated metaphyseal/endosteal cell pool. Compared to skeletal cells isolated from neonatal bone, metaphyseal/endosteal bone contained a similar percentage of skeletal stem cells, whereas the number of multipotent pre-progenitors was significantly higher. A possible explanation for this observation is that during the isolation of neonatal SSPCs, we used total bone including the growth plate, which contains CD105-expressing chondrocytes (19, 26). In addition, this difference could also be due to the fact that the metaphyseal/endosteal region of young-adult bone is characterized by active bone formation and therefore requires a sufficiently high number of potent progenitor cells. Finally, our data confirm earlier studies that showed the presence of multipotent progenitors in the bone metaphysis/endosteum based on the expression of Gli1, Grem1, LepR, PDGFRα and PDGFRβ and Nestin-GFP (7–13). Because the putative SSPC markers described by Chan et al. may not label all SSPC subpopulations in adult bone, future studies are necessary to determine the functional overlap of the metaphyseal/endosteal SSPCs described in our study with those identified using the single-fluorescent reporter models described above.

Our flow cytometry analysis also revealed a considerable amount of hematopoietic and/or vascular cells in both freshly isolated and culture-expanded metaphyseal/endosteal bone cells that may interfere with downstream *in vitro* analysis. Therefore, to enrich for skeletal cells, we depleted CD45, CD31 and Ter119-expressing cells using MACS. Compared to conventional FACS, cell sorting using magnetic antibody-coated nanoparticles allows for faster processing speed and easier handling, which better preserves cell viability and thus results in a greater cell yield (31). Moreover, as the MACS setup can be readily used in a sterile flow cabinet, it limits cell culture contamination. Finally, there is no need for specialized cell sorting equipment, thereby reducing the overall cost significantly. Together, we present a fast, highly feasible and cost-effective method for isolating SSPCs from metaphyseal/endosteal young-adult bone.

It is well-accepted that SSPCs include a population of cells with self-renewing potential. To assess whether metaphyseal/endosteal SSPCs possess similar characteristics *ex vivo*, we analyzed their ability to serially form colonies at low-seeding density, which is considered as the golden standard for investigating *in vitro* self-renewing properties (32, 33). We noticed that metaphyseal/endosteal SSPCs displayed a higher primary colony efficiency compared to SSPCs from neonatal mice. On the other hand, secondary colony formation was similar between metaphyseal/endosteal and neonatal SSPCs, which is in line with our FACS observation that the percentage of putative stem cells was comparable between the two SSPC pools. Whether the difference in primary CFU efficiency is caused by the presence of other, still unknown progenitor populations with colony-forming potential within metaphyseal/endosteal bone requires further research. In addition to their self-renewing potential, metaphyseal/endosteal SSPCs have the ability to differentiate into the osteogenic, adipogenic and, to a lesser extent, chondrogenic lineage *in vitro*. Of note, we observed that expansion of SSPCs in hypoxia markedly improved cell growth and differentiation potential, which is in line with previous reports (28–30). This beneficial effect is likely caused by more closely mimicking the oxygen levels within the bone marrow (34). Possibly, using a media formulation that better matches the metabolic microenvironment *in vivo* might further enhance SSPC properties, but this remains an outstanding question.

At first sight, our observations of SSPC differentiation *in vitro* did not fully reflect the study by Chan et al, who described that SSCs and (pre-)BCSPs only give rise to bone, cartilage and stroma, but not adipocytes. However, the adipogenic potential of these cells was only inferred from clonal-lineage studies using Rainbow mice, and was not investigated using *in vivo* transplantation with sorted cells or *in vitro* differentiation assays. Moreover, recent studies clearly demonstrate the presence of Sca1^-^CD51^+^PDGFRα^-^PDGFRβ^-^ and Sca1^-^CD51^+^PDGFRα^+^PDGFRβ^-^ skeletal progenitors in the bone metaphysis that can give rise to marrow adipocytes, although it remains to be determined whether these cells show overlapping features with the metaphyseal/endosteal SSPCs described here (12). On the other hand, *in vitro* assays do not always reflect the *in vivo* situation. For example, mesenchymal stromal cells are very sensitive to tissue level elasticity in the microenvironment and can commit to specific lineages accordingly (35). The use of plastic culture dishes may thus bias metaphyseal lineage allocation towards adipogenesis, although this hypothesis requires further testing.

Taken together, we propose a standardized protocol to isolate and culture SSPCs from the long bone metaphysis/endosteum of young-adult mice. The enzymatic digest and isolation procedure are fast, reliable and cost-effective, and the MACS-dependent enrichment of skeletal cells after expansion is highly efficient. Based on the expression of known surface markers (19), we show that metaphyseal and endosteal bone contains skeletal stem and multipotent progenitor cells that display *in vitro* self-renewal and multipotency, making them a suitable cell culture model to study skeletal disorders that are associated with altered SSPC behavior. Possible examples include, but are not limited to, age-related or postmenopausal osteoporosis, or bone dysfunction in chronic diseases such as obesity, diabetes or inflammation (36–39). Moreover, the here-established isolation protocol allows for the comparison of metaphyseal/endosteal SSPCs with SSPCs from other niches, such as periosteum and bone marrow stroma, which will help better understand SSPC biology.

## Data availability statement

The original contributions presented in the study are included in the article/**Supplementary material**. Further inquiries can be directed to the corresponding author.

## Ethics statement

The animal study was reviewed and approved by Institutional Animal Care and Research Advisory Committee of the KU Leuven.

## Author contributions

SL, GC and SS conceptualized and designed the study. SL, IS, and SS acquired data. SL, GC and SS performed analysis and interpretation of the data. SL, GC and SS wrote the manuscript. All authors contributed to the article and approved the submitted version.

## Funding

GC acknowledges funding from the Research Foundation-Flanders (FWO: G.0B3418 and G0C5120N) and the KU Leuven (C24/17/077). SL was funded by a PhD fellowship strategic basic research from the FWO (1S46318N). SS is a postdoctoral fellow from the FWO (12H5917N).

## Acknowledgments

The authors wish to thank Karen Moermans and Sophie Torrekens for technical assistance.

## Conflict of interest

The authors declare that the research was conducted in the absence of any commercial or financial relationships that could be construed as a potential conflict of interest.

## Publisher’s note

All claims expressed in this article are solely those of the authors and do not necessarily represent those of their affiliated organizations, or those of the publisher, the editors and the reviewers. Any product that may be evaluated in this article, or claim that may be made by its manufacturer, is not guaranteed or endorsed by the publisher.

## Supplementary material

Supplementary Figure 1 CD45, CD31 and Ter119 expression in metaphyseal/endosteal and neonatal cell population with and without MACS-mediated depletion. (A, B) Contour plots showing the gating strategy used for flow cytometry-based quantification of the percentage of CD45^-^CD31^-^Ter119^-^ cells in cultured metaphyseal/endosteal cells without (A) and with (B) MACS-mediated depletion of CD31^+^/CD45^+^/Ter119^+^ cells (n=3). (C, D) Contour plots showing the gating strategy used for flow cytometry-based quantification of the percentage of CD45^-^CD31^-^Ter119^-^ in cultured neonatal cells without (C) and with (D) MACS-mediated depletion of CD31^+^/CD45^+^/Ter119^+^ cells (n=3). Data are shown as mean ± SD.

Supplementary Figure 2 MACS depletion does not alter CD105 and CD200 expression in cultured metaphyseal/endosteal, but is beneficial for osteogenic capacity (A) Table showing the flow cytometry-based quantification of the percentage of CD105 and CD200 expression in cultured metaphyseal/endosteal cells with or without MACS-mediated depletion of CD31^+/-^/CD45^+/-^/Ter119^+/-^ cells (n=3). (B) Alizarin Red staining at day 21 of osteogenic differentiation of metaphyseal/endosteal SSPCs with or without MACS-mediated depletion. Data are shown as mean ± SD.
