# Supplementary figures and images for: Isolation and in vitro characterization of murine young-adult long bone skeletal progenitors
Source: Front Endocrinol (Lausanne). 2022 Aug 1;13:930358. doi: 10.3389/fendo.2022.930358 (PMC9376626; doi:10.3389/fendo.2022.930358)

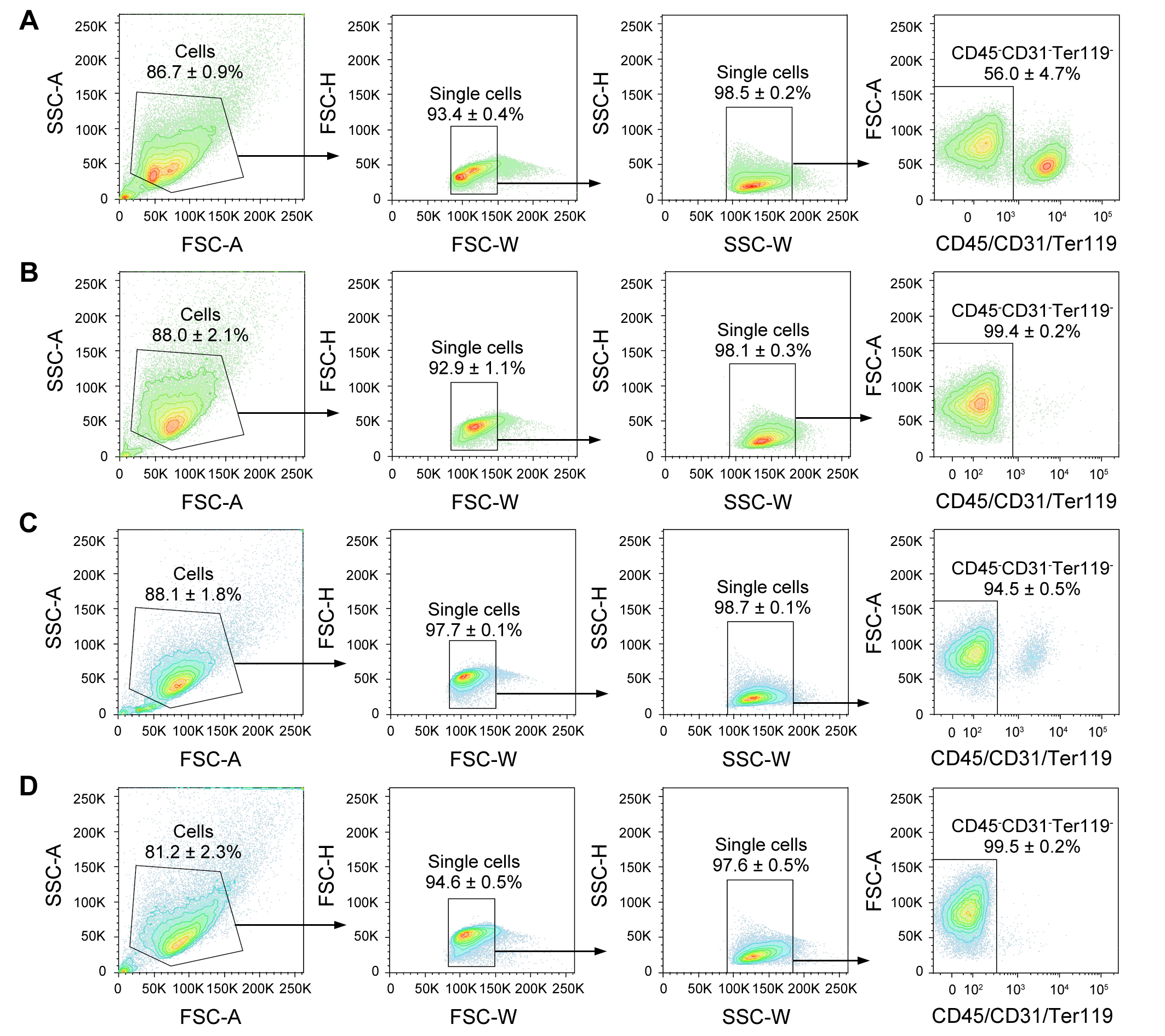

Supplement: Supplementary Figure 1 — CD45, CD31 and Ter119 expression in metaphyseal/endosteal and neonatal cell population with and without MACS-mediated depletion. (A, B) Contour plots showing the gating strategy used for flow cytometry-based quantification of the percentage of CD45-CD31-Ter119- cells in cultured metaphyseal/endosteal cells without (A) and with (B) MACS-mediated depletion of CD31+/CD45+/Ter119+ cells (n=3). (C, D) Contour plots showing the gating strategy used for flow cytometry-based quantification of the percentage of CD45-CD31-Ter119- in cultured neonatal cells without (C) and with (D) MACS-mediated depletion of CD31+/CD45+/Ter119+ cells (n=3). Data are shown as mean ± SD. [file Image_1.tif]

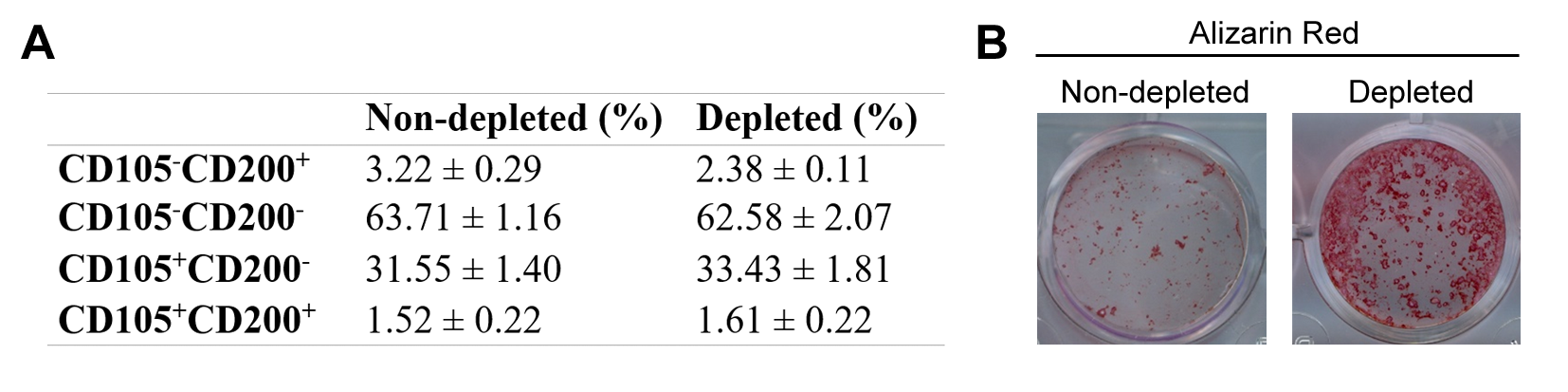

Supplement: Supplementary Figure 2 — MACS depletion does not alter CD105 and CD200 expression in cultured metaphyseal/endosteal, but is beneficial for osteogenic capacity (A) Table showing the flow cytometry-based quantification of the percentage of CD105 and CD200 expression in cultured metaphyseal/endosteal cells with or without MACS-mediated depletion of CD31+/-/CD45+/-/Ter119+/- cells (n=3). (B) Alizarin Red staining at day 21 of osteogenic differentiation of metaphyseal/endosteal SSPCs with or without MACS-mediated depletion. Data are shown as mean ± SD. [file Image_2.tif]
